# Flexure-based Roll-to-roll Platform: A Practical Solution for Realizing Large-area Microcontact Printing

**DOI:** 10.1038/srep10402

**Published:** 2015-06-03

**Authors:** Xi Zhou, Huihua Xu, Jiyi Cheng, Ni Zhao, Shih-Chi Chen

**Affiliations:** 1 Department of Mechanical and Automation Engineering, The Chinese University of Hong Kong, N.T.,Hong Kong SAR, China; 2 Department of Electronic Engineering, The Chinese University of HongKong, N.T., Hong Kong SAR, China

## Abstract

A continuous roll-to-roll microcontact printing (MCP) platform promises large-area nanoscale patterning with significantly improved throughput and a great variety of applications, e.g. precision patterning of metals, bio-molecules, colloidal nanocrystals, etc. Compared with nanoimprint lithography, MCP does not require a thermal imprinting step (which limits the speed and material choices), but instead, extreme precision with multi-axis positioning and misalignment correction capabilities for large area adaptation. In this work, we exploit a flexure-based mechanism that enables continuous MCP with 500 nm precision and 0.05 N force control. The fully automated roll-to-roll platform is coupled with a new backfilling MCP chemistry optimized for high-speed patterning of gold and silver. Gratings of 300, 400, 600 nm line-width at various locations on a 4-inch plastic substrate are fabricated at a speed of 60 cm/min. Our work represents the first example of roll-to-roll MCP with high reproducibility, wafer scale production capability at nanometer resolution. The precision roll-to-roll platform can be readily applied to other material systems.

Roll-to-roll (R2R) printing technology has been the driving force for the development of many flexible electronic and photonic devices. It shows tremendous advantages in terms of cost and throughput when compared with lithographic fabrication processes for conventional inorganic semiconductor devices. However, a frequently held misconception about the R2R process is that it produces devices of lower resolution and quality. This is not necessarily true if contact printing techniques such as microcontact printing (MCP)^1^,^2^,^3^,^4^,^5^,^6^,^7^,^8^,^9^ or nanoimprint^10^,^11^,^12^,^13^ are implemented to properly designed R2R platforms. In fact, the printing resolution is potentially better than that of lithographic processes as contact printing is not limited by the diffraction of light. In the past decade, complex high resolution patterns of tens of nanometers have been repeatedly demonstrated by the aforementioned techniques at laboratory scale^2^,^12^. However, there are a number of reasons why it is not practical to resort to direct scale-up and apply these techniques to the present state-of-the-art R2R systems, e.g. gravure printing and flexographic printing, which have approximately 20 micron print resolution. First, these systems are designed with conventional mechanical components and bearings; hence lack the required nanometer level repeatability and accuracy. In addition to the unmatched mechanical performance, the related chemical formulations and process requirements, such as the temperature, also prevent the direct adaptation of contact printing technologies to a high-throughput R2R platform. To be more specific, for MCP, the thiol-based chemistry requires an etching time as long as 20 minutes due to the weak protection of self-assembled monolayer (SAM) and the use of mild etchant. For nanoimprint, the high temperature and plastic deformation steps preclude the high-throughput application. Additive MCP processes, e.g. two-step press microcontact printing (TSP-MCP), was developed to transfer dry ink from PDMS to a substrate^14^. However, the inking process is slow and adhesion of the ink to a hydrophobic surface could be challenging. Also, microscale cracks can be easily developed during the transfer and annealing processes of the nanoparticles.

In this work, we developed a flexure-based R2R system (shown in Fig. 1) that achieved nanometer level repeatability and precision in all six axes and realized high-resolution and high throughput printing by adapting MCP to the R2R platform. Flexures, which rely on the compliance of their structure to generate smooth and controlled motions, provide many advantages over traditional mechanical linkages/joints for precision motion guidance^15^,^16^,^17^. MCP was chosen over nanoimprint and other contact printing techniques as it is inherently a faster process (molecular ink transfer is instantaneous) and allows a wider range of material choices^3^,^4^,^5^,^6^,^7^,^8^,^9^, including but not limited to, metals^5^,^6^, bio-molecules^7^,^8^ and quantum dots^9^. However, to transfer nanoscale patterns from an elastomer stamp to a 4-inch flexible web (substrate), the required positioning precision for the control of parallelism between rollers needs to be within 500 nm, and the contact forces to be within 0.05 N over a large area. Moreover, new MCP recipes are needed for the R2R printing process at high speed. In the following sections, we report (1) the design and characterization of the flexure-based R2R platform, and (2) the development of a new positive MCP process. Gold and silver electrodes and optical gratings are fabricated to demonstrate the scalability and resolution of the new R2R MCP process.

## Results and Discussion

### Flexure-Based Precision R2R Machine

**Figure 1:**
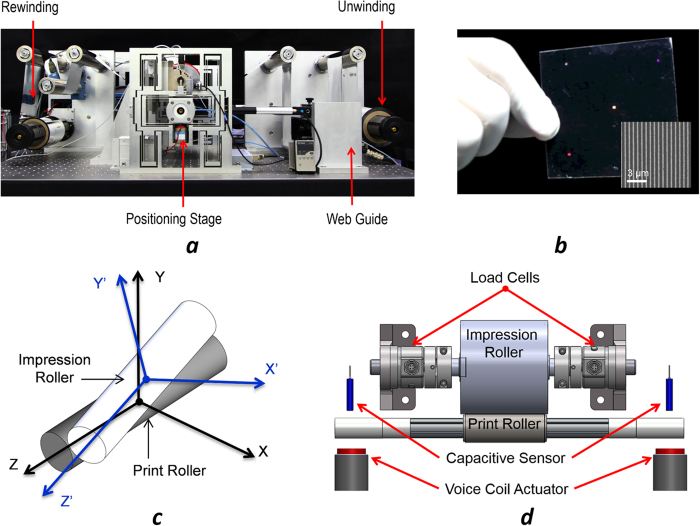
(**a**) Flexure-based R2R printing system. (**b**) Image of optical gratings (linewidth: 300–600 nm) printed on a 4-inch flexible PET substrate. (**c**) Illustration of misalignments between impression roller and print roller. (**d**) Front view of the print module, where two ends of the print roller are supported by the flexure stages with integrated voice coil actuators.

In a R2R system, printing occurs when the web goes through the impression and print roller with controlled pressure, tension and alignment. However, conventional R2R platforms lack alignment features to compensate the “yaw” and “roll” errors (that is, rotational errors in *θ*_*Y*_ and *θ*_*X*_ respectively as in Fig. 1c) between the impression and print roller during printing, which leads to non-uniform distribution of pressure and pattern distortions. More importantly, in order to achieve uniform printing results at nanoscale, it is necessary to have precise control of all six degrees of freedom of the print roller. Despite the fact that a few precision R2R printing systems were developed recently with reasonable success^18^,^19^, and that the contact mechanism of the MCP based printing process was studied^19^,^20^,^21^, an R2R machine that meets all design requirements has never been demonstrated.

**Figure 2:**
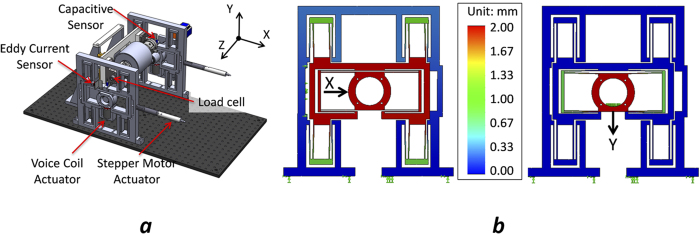
(**a**) CAD model of the printing module, where print roller is supported by two monolithic flexure stages. (**b**) Simulated results of the decoupled X and Y motions generated by the flexure stage. The color bar represents the magnitude of the displacement.

Flexure mechanisms inherently possess nanometer level repeatability at the expense of a large workspace to footprint ratio and coupled degrees of freedom for positioning. We propose a new design that exploits the benefits while minimizing the system footprint with decoupled position control. As shown in Fig. 1, our R2R system consists of four sub-modules: Unwinding module, printing module, web guide module, and rewinding module, where ultra-precision printing is enabled by the critical printing module. Fig. 1d illustrates the detailed design of the printing module. To ensure stable operation, air bearings are used to guide the motions of the print roller. The impression roller is mounted on a Z-stage with integrated load cells at its two ends to monitor the printing forces. The print roller is supported by two monolithic flexure stages that provide decoupled X and Y motion guidance with nanometer repeatability and precision. The motion decoupling is achieved through the combination of multiple folded beam structures (Fig. 2). Since motions are generated from the elastic deformation of the monolithic compliant stage, errors from backlash or assembly are completely eliminated. The flexure stages are driven by voice coil actuators in the Y direction and high-resolution linear actuators in the X direction. Their positions are monitored by two pairs of capacitance probes and eddy current sensors. These sensors provide high bandwidth (15 kHz) real-time position feedback to implement closed-loop control in order to achieve constant parallelism between the impression and print rollers. Fig. 2a illustrates the layout of the monolithic flexure stages in the printing module; Fig. 2b presents simulated results of the flexure stage subjected to an input displacement of 2 mm in X direction and -2 mm in Y direction. It can be seen that cross-axis motion coupling errors are completely removed by the flexure mechanism. Further details of the R2R machine are described in the Method section.

### Characterization of the R2R Machine

The flexure stages were designed and optimized through a parametric model^22^ that precisely predicts their static and dynamic mechanical properties, including natural frequency, mode shapes, and damping coefficients. Furthermore, analytical models have been developed to predict pattern distortion based on the stamp/roller interaction. These parameters, after validated experimentally, were programmed into the Multi-Input, Multi-Output (MIMO) PID controller to position the print roller with nanometer level precision in different axes.

We demonstrate the precise force and position control capability of the R2R machine through two sets of experiments. These experiments are important as print forces ranging from 0 – 20 N are required to create patterns of various scales, e.g. 10 s nm to 10 s microns. Note that the contact force should be minimized when the stamp and the substrates are perfectly flat. In practice, to ensure conformal contact without collapsing the nanoscale features on the stamp, uniform and precise force control around 3 ± 0.025 N is critical based on our experiments.

**Figure 3:**
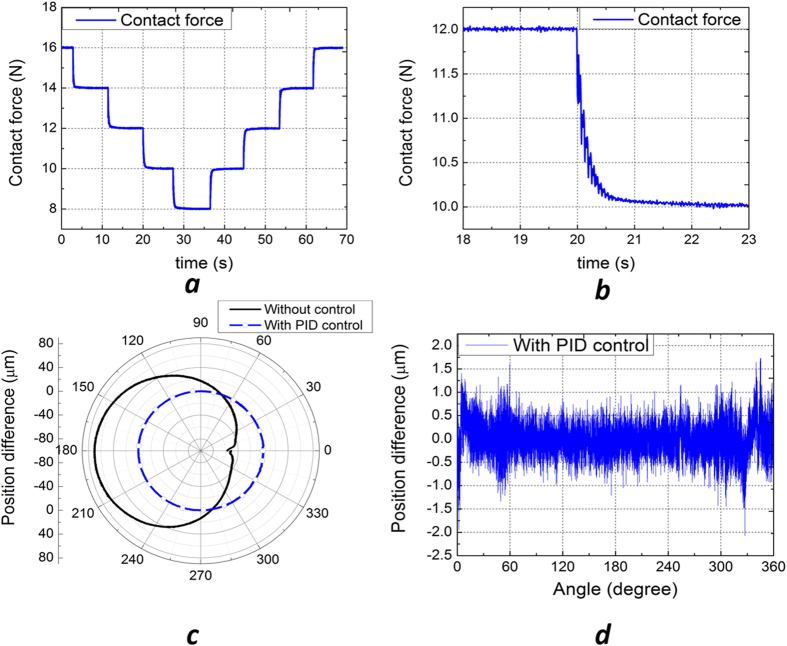
(**a**) Contact force experiment for 2 N force steps. (**b**) A zoom-in view of a single step showing the precise force control with no overshooting forces. (**c**) Position differences of the print roller over a revolution. (**d**) Position difference with PID control.

In the first printing force experiment, the flexure stages, i.e., the print roller, were commanded to move down and up to demonstrate 2 N force steps, Fig. 3a,b. It is worthwhile to note that the overshooting of the print roller was completely removed by the PID controller. The roll angle of the print roller, namely, parallelism, was controlled within ±2 μrad throughout this experiment. In the second experiment, we recorded position differences between the two ends of the print roller for a full revolution (360^o^) with 15 N print force. Note that perfect parallelism is achieved when the position difference is zero. The results for both open-loop and closed-loop data were as shown in Fig. 3c,d. A number of factors were attributed to the open loop error which caused a 10 N print force variation over a revolution: (1) the roller eccentricity due to manufacturing error, and (2) the disconnected gap from the stamp wrapped around the print roller. In the case where the controller was activated, the roller eccentricity was completely removed and the position difference was controlled within 500 nm (~2 μrad roll angle), hence satisfying the precision requirements for large area MCP applications. In other words, a new high level of precision was firstly demonstrated on a R2R system. A video demonstrating the fully automated printing process is provided in the Supporting Information.

### Positive Microcontact Printing

**Figure 4:**
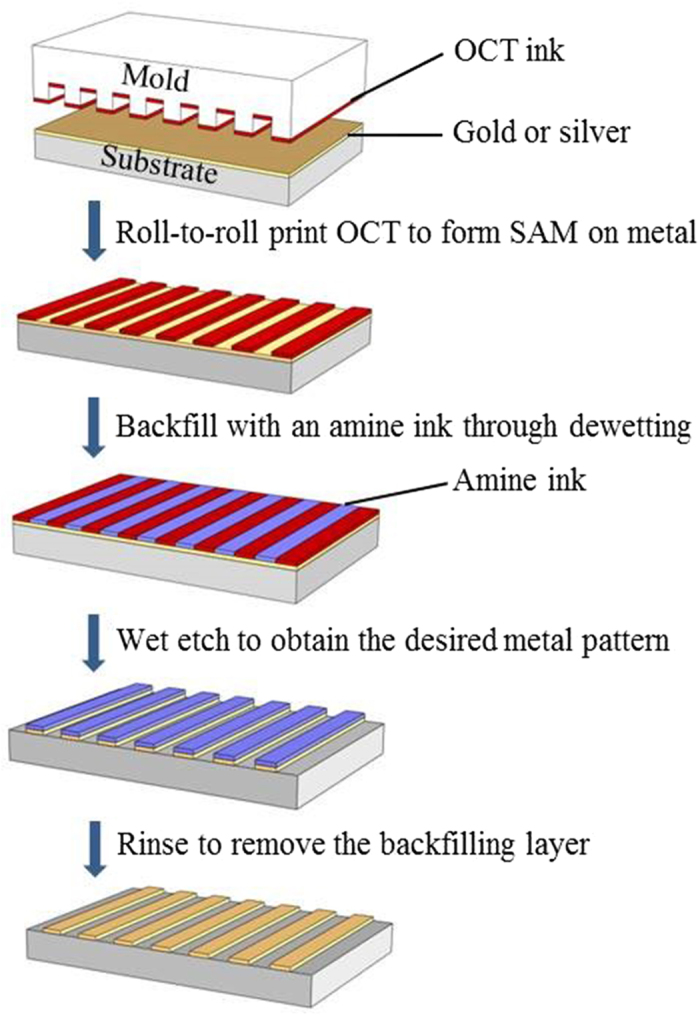
Schematic illustration of the positive microcontact printing.

To fully realize the benefits of high-speed printing on a large area achieved by the R2R system, we have developed a positive MCP procedure for the fabrication of micro- and nano-scale metal patterns. This procedure features a two-step surface-directed patterning process combining the use of thiol self-assembled monolayer (SAM) and aromatic amine backfilling ink, with the latter being the key to a fast, selective and highly reproducible metal patterning process. The detailed positive MCP procedure is illustrated in Fig. 4.

Initially, a patterned PDMS mold was inked with ethanolic 1-octadecylthiol (OCT) and brought into contact with a metal surface. Due to the chemical interaction between the thiol and metal (Au or Ag), a patterned OCT SAM was generated on the metal surface, rendering the surface partial hydrophobic (with SAM coverage) and partial hydrophilic (without SAM coverage)^23^. In the following step, an aromatic amine backfilling ink was cast onto the metal surface. During drying, the ink was repelled by the hydrophobic surface region to leave a thin aromatic amine layer covering only the hydrophilic metal area. This layer plays a crucial role in protecting the metal during the following etching process. A potassium iodide-iodine-based etchant was then applied to the whole metal surface to remove the area covered by SAM, thus forming a positive replica of the original master. (As such, the whole process is referred to as “positive microcontact printing^24^”).

The formulation of the backfilling ink is a key processing parameter to enable high throughput positive MCP. The backfilling molecules should provide a moderate adhesion to the metal such that they can protect the metal during the etching process while being easily removed in the final rinsing step. We selected a group of ionic aromatic amine molecules as the backfilling material, where the weak coordination between the nitrogen and gold (or silver) atoms was exploited to meet the adhesion requirement. Small molecules, rather than polymers, were chosen to ensure a low viscosity of the backfilling ink. (See Supporting Information Table S1 for the results obtained from polymer based backfilling inks). Table 1 lists the results of positive MCP for five backfilling molecules with mono-, di- and tri- amino groups, respectively. The hexagonal mesh patterns were fabricated on PET substrates. Best results were yielded by the compounds 2 and 3 with diamino groups where the backfilling layer was well defined in the hydrophilic areas and good protection was rendered to the metal during etching. After the final rinsing step, the amine layer was removed completely, leaving metal patterns with sharp edges and clean surface. By contrast, the monoamine backfilling layer (based on compounds 4 and 5) appeared to be partially destroyed during etching, resulting in non-uniformity thinning of the metal patterns. Furthermore, the triamine (compound 1) backfilling layer exhibited an etching resistance that spreaded around the hydrophobic-hydrophilic boundaries. As a result, the edges of the metal pattern were smeared out after the etching and rinsing procedures.

According to Table 1, the 3,6-diaminoacridine hydrochloride (compound 2) in a mixture of ethanol and diacetone alcohol (DAA) (9:1, v/v) was selected as the backfilling ink to fabricate micro- and nano-scale metal patterns. The combination of ethanol and DAA was chosen due to its low-toxicity, moderate drying speed (three seconds under a nitrogen stream) and ability to fully dissolve the backfilling molecules, all of which are crucial for realizing large-scale R2R production in ambient conditions. For the selective etching process, we chose an aqueous etchant mixture of potassium iodide and iodine with its mixing ratio determined based on the type of metal (Au or Ag) to be etched (See Methods). Our experiments demonstrated that a 40 nm OCT SAM covered gold film could be completely etched away in six seconds, or four seconds for silver of the same thickness. Such a high etching speed would help pave the way for adapting the positive MCP process to a high throughput R2R system.

To summarize, there are several reasons why the positive MCP protocol is well suited for our ultrahigh precision R2R system. Firstly, MCP is capable of producing structures with submicron dimensions (*2*). Secondly, introducing backfilled aromatic amine molecules as etch resist allows the use of aggressive etchants, e.g. potassium iodide-iodine system. This significantly shortens the time of metal etching, the rate-limiting step of the R2R process. By contrast, the conventional microcontact printing processes which rely on thiol SAM as the etch resist necessitate a mild etching process for a much longer etching time (~17 minutes for 50 nm thickness gold, ~7 minutes for 20 nm thickness silver)^23^,^25^. Lastly, the backfilling process produces a uniform and robust protection layer with well-defined edges, an extraordinary advantage for producing nanopatterns with low defect density over a large area. We note that in a multi-layer printing process, the etching step of the positive MCP may cause damage to the underlying layers. For these applications, additive printing processes, such as TSP-MCP^14^ may be adapted to the R2R platform to achieve better results.

### Microscale and Nanoscale Metal Patterns

**Figure 5:**
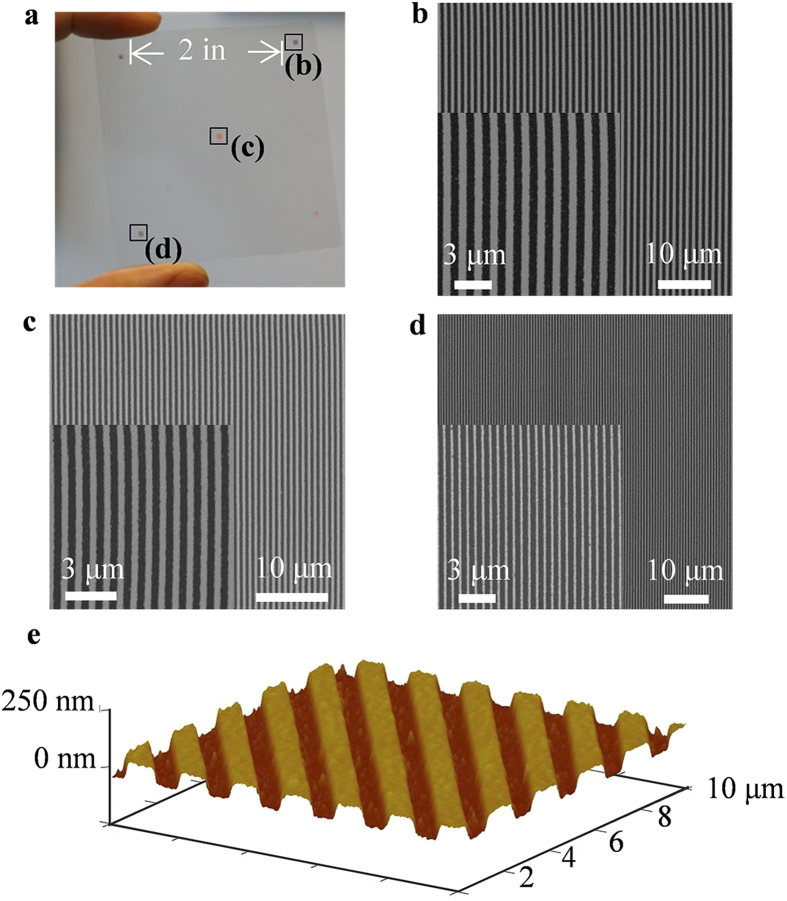
Gold nanograting patterns on flexible PET substrates fabricated by R2R MCP. (**a**) Photo of the gold-nanograting arrays. The light diffraction from the periodic gratings can be observed clearly. (**b**) SEM images of the nanogratings with 600 nm line width, 600 nm spacing. (**c**) 400 nm line width, 400 nm spacing. (**d**) 300 nm line width, 300 nm spacing. (**e**) 3-D AFM image of 600 nm line width gratings.

Nanoscale gold optical gratings were fabricated on flexible PET substrates using the R2R MCP system with a composite hard 184-PDMS mold. The rolling speed was 0.01 m s^−1^ and the printing pressure was 3 N. Five grating patterns with line width and spacing of 300 nm, 400 nm and 600 nm, respectively, were produced in one printing pass on a 4-inch substrate. As shown in Fig. 5a, the patterns are up to 3 inches apart, demonstrating that the system is capable of large-area nanopatterning. The patterns reveal the high uniformity of the gold lines as well as the cleanness of the substrate after etching, as the SEM and AFM images shown in Fig. 5b–e.

**Figure 6:**
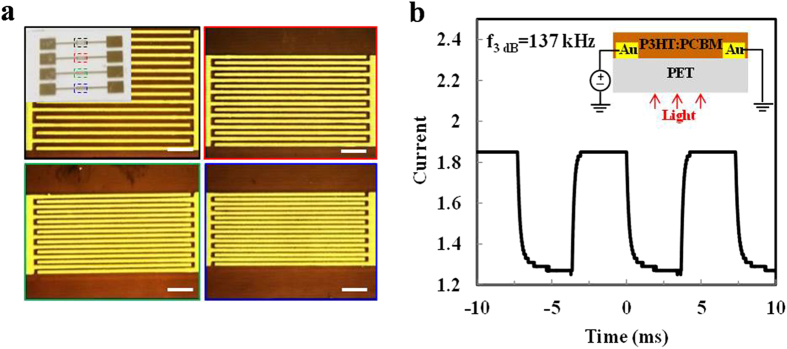
Photodetector using gold interdigitated electrodes on flexible PET substrates fabricated by R2R MCP. (**a**) Optical images of gold interdigitated microelectrodes with different electrode spacings (L): black dashed box, L = 15 μm; red dashed box, L = 10 μm; green dashed box, L = 5 μm; blue dashed box, L = 3 μm (Scale bar = 100 μm). The inset shows the electrodes array. (**b**) Temporal photocurrent response of the photodetector based on R2R microcontact printed gold interdigitated electrodes. The inset shows the device structure.

To further demonstrate the practical use of the R2R printed metal patterns, we fabricated microscale gold and silver electrodes on flexible PET substrates. In this case a 184-PDMS mold was used, and the rolling speed and printing pressure were increased to 0.02 m s^−1^ and 15 N, respectively. As shown in the inset of Fig. 6a, gold interdigitated electrodes with channel lengths of 15 μm, 10 μm, 5 μm and 3 μm were obtained simultaneously through one printing pass. A planar photoconductive photodetector (inset of Fig. 6b) was fabricated using the gold interdigitated electrodes. The photosensitive layer was formed by a bulk heterojunction of poly(3-hexyl thiophene) (P3HT) and [6-phenyl C61-butyric acid methyl ester (PCBM). The photodetector with 3 μm channel length demonstrated fast temporal response, as in Fig. 6b. Silver micro-patterns were also fabricated and used as the transparent electrodes for organic photovoltaic cells (Supporting Information, Fig. S1).

## Conclusions

We have developed a flexure-based R2R platform that enables six-axis active control of the print module at nanometer level precision. High-throughput, large area continuous roll-to-roll printing at 100 s nanometer resolution is realized through adapting a positive MCP protocol with new inking and etching chemistry to the R2R platform. Uniform micro- and nano-scale gold and silver electrodes and optical gratings over a 4-inch flexible substrate have demonstrated the efficacy of the positive MCP recipes as well as the precision of the R2R platform. Being one of the most versatile techniques for nanoscale patterning, MCP is traditionally prevented from a direct scale-up for its demand for extreme precision with multi-axis positioning. Our flexure based design proves to overcome such barrier. Although the study focuses only on the nano-patterning of metals, the R2R platform can be readily applied to other materials, as long as the corresponding MCP process has a R2R compatible time scale. The kinetic control and surface chemistry of the MCP process should be carefully tuned for each material system. Furthermore, by including an additional set of four-axis flexure stages and cameras for real-time position monitoring, multi-layer registration can be realized with MCP or other transfer printing techniques on our R2R system.

## Methods

### R2R machine

The lateral position of the web is controlled by the web-guiding module, as in Fig. 1. The module consists of four rollers, with two of the upper rollers affixed to a rotary stage controlled by a servo motor. The actual position of the web is detected by an infrared edge sensor. When the rollers in the upper frame rotated, the unbalanced friction force causes the web to move in the lateral (Z) direction. This arrangement ensures the web path to be corrected within a short distance with minimized stress in the web. The web is driven by a stepper motor connected to the print roller shaft with an elastomeric coupler. Closed-loop web tension control is achieved through the use of a magnetic particle clutch and brake as well as tension sensor, where optimal printing results can be achieved when the web is at 10% tensile strain^26^. All data acquisition and controls are conducted on a PC and NI DAQ system (PXI 6356).

To adapt the MCP process to the R2R system, common substrates are replaced with metal (gold and silver) coated PET webs. For stamp preparation, PDMS stamps are fabricated by spin coating procedure before being attached to a glass tube which is then fixed to the motor-driven shaft. To reduce the external disturbances such as vibration, the R2R platform was constructed on an air table (Smarttable Top/S-2000 isolators, Newport) so as to reduce the ground vibration to within 40 nm.

The monolithic flexure stages were fabricated from a 1 inch thick aluminum 7075 plate through electrical discharge machining. Actuation and sensing components used on the flexure stage include: (1) voice coil actuators (NCC03-15-050-2X, H2W), (2) linear actuators (M230.10S, PI), (3) capacitance probes (C30/CPL290, Lion Precision), and (4) eddy current sensors (EX-416V, Keyence).

### Chemicals and reagents

All commercial reagents were obtained from Aldrich Chemical Co. and used without further purification unless otherwise specified. Hard PDMS components: (7.0-8.0% vinylmethylsiloxane)-dimethylsiloxane copolymer (VDT-731), platinum-divinyltetramethyldisiloxane (SIP6831.2), 1,3,5,7-tetravinyl-1,3,5,7-tetramethylcyclotetrasiloxane (SIT7900.0), and (25-30% methylhydrosiloxane)-dimethylsiloxane copolymer (HMS-301) were purchased from Gelest. The soft PDMS elastomer kit (Sylard 184) was ordered from Dow corning. The alkanethiol solution was prepared by dissolving 1-octadecylthiol in ethanol to obtain a concentration of 5mM. The amine backfilling solution was prepared by dissolving 10 mg ml^−1^ 3,6-diaminoacridine hydrochloride in a mixture of ethanol and diacetone alcohol and then immediately filtered through 0.22 μm syringe filters before use.

### Metal etchant

A potassium iodide/iodine mixture solution was selected as the etchant of gold and silver due to its high reactivity and low toxicity. For gold etching, a commercial potassium iodide/iodine formula (Gold Etchant TFA) was used. For the more reactive silver, an aqueous solution of 1.2 M potassium iodide mixed with 0.03 M iodine was prepared.

### Substrate preparation

The PET substrate was obtained from Dupont and had a thickness of 265 gauge. The substrates were ultrasonically cleaned with acetone, IPA and deionized water, and finally blow-dried with nitrogen. A 40-nm gold or silver layer was then sputter deposited on the PET substrate.

### PDMS mold

Sylard 184 was used for fabricating molds at micrometer-scale. The 184-PDMS molds were fabricated by spin coating the prepolymer mixture on photolithographically prepared 4- inch silicon masters with micrometer-scale patterned photoresist. The features of the silicon master were replicated on the PDMS mold surface after curing, and the thin PDMS mold was peeled off the master, wrapped around a 36 mm diameter cylindrical glass roller and inked with a 5 mM ethanol solution of OCT.

Composite molds composed of a hard PDMS layer supported by a soft 184 PDMS layer were used to replicate nanogratings on the silicon master. The 4-inch silicon master with five periodic grating patterns with line width and spacing of 300 nm, 400 nm and 600 nm, respectively, was fabricated by e-beam lithography and dry etching. The composite PDMS molds were prepared according to the literature procedure^27^,^28^. After curing, the PDMS mold was peeled away, wrapped around the cylindrical roller and inked with the OCT solution prior to use. After printing, the PDMS tube can be removed from the print roller and cleaned by rinsing with ethanol and blow-drying with nitrogen.

### Positive microcontact printing

The OCT ink on the cylindrical PDMS mold was R2R printed across the gold surface on a PET substrate to generate OCT SAM. For micrometer pattern fabrication, the rolling speed was 0.02 m s^−1^, and the printing pressure was 15 N; for nanometer patterns, the rolling speed was 0.01 m s^−1^, and the printing pressure was 3 N. Afterwards, 3,6-diaminoacridine hydrochloride in a mixture of ethanol and DAA (9:1, v/v) solution was cast on the patterned substrate to backfill the rare gold areas. Upon the completion of dewetting and drying of the backfilling ink under a nitrogen stream , the sample was immersed in gold etchant for six seconds to selectively remove the gold regions covered by OCT SAM, and the positive replica of the silicon master was obtained after rinsing away the backfilling layer with ethanol and DI water.

### Analysis techniques

The samples were investigated by optical microscopy (Micromanipulator 7000), and desktop scanning electron microscopy (SEM) (Phenom ProX). The AFM image was taken with a VEECO Dimension^TM^ 3100.

### Device fabrication

The photodetector was fabricated by spin coating a 40 mg ml^−1^ PCBM:P3HT solution in dichlorobenzene on top of the R2R printed gold interdigitated electrodes on PET substrate (Fig. 6b). Measurements were carried out after annealing the device at 155 °C under a nitrogen atmosphere for 15 minutes. A Newport LQD laser diode (635 nm emission) driven by an Agilent 33220A function generator was used as the light source to provide square wave modulated illumination.

## Additional Information

**How to cite this article**: Zhou, X. *et al.* Flexure-based Roll-to-roll Platform: A Practical Solution for Realizing Large-area Microcontact Printing. *Sci. Rep.* **5**, 10402; doi: 10.1038/srep10402 (2015).

## Supplementary Material

Supplementary Information

Supplementary Information

## Figures and Tables

**Table 1:**
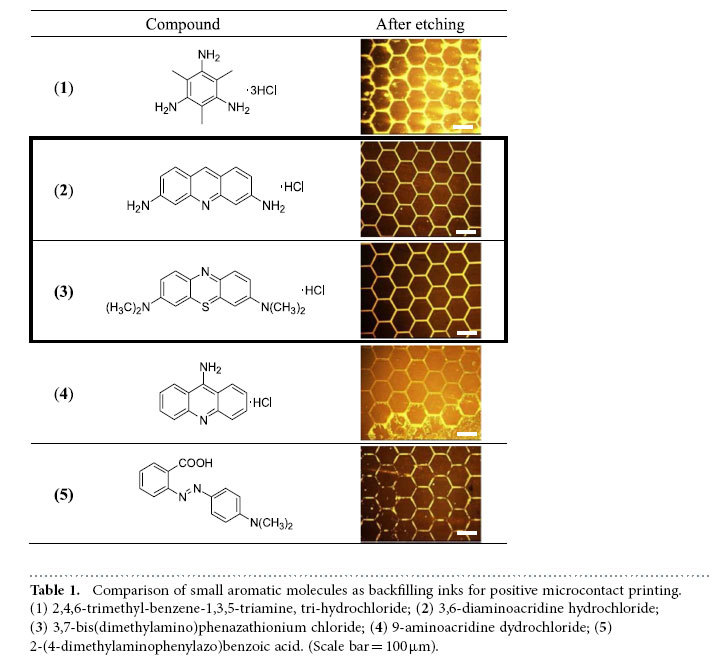
Comparison of small aromatic molecules as backfilling inks for positive microcontact printing.
