# Supplementary material for: Flexure-based Roll-to-roll Platform: A Practical Solution for Realizing Large-area Microcontact Printing
Source: Sci Rep. 2015 Jun 3;5:10402. doi: 10.1038/srep10402 (PMC4603702; doi:10.1038/srep10402)
Supplement: Supplementary Information [file srep10402-s1.pdf]

Supporting Information for

Flexure-based Roll-to-roll Platform: A Practical Solution for Realizing Large-area Microcontact Printing

Xi Zhou[1, ‡], Huihua Xu[1, ‡], Jiyi Cheng[1], Ni Zhao[2,*], and Shih-Chi Chen[1,*]

[1]Department of Mechanical and Automation Engineering, The Chinese University of Hong Kong, N.T., Hong Kong SAR, China; and [2]Department of Electronic Engineering, The Chinese University of Hong Kong, N.T., Hong Kong SAR, China.

[*]Corresponding authors

Email: scchen@mae.cuhk.edu.hk; nzhao@ee.cuhk.edu.hk

1. Polymer based backfilling inks

Polymers including poly(methyl methacrylate) (PMMA)[1] and polyethylenimine (PEI) were examined as the backfilling ink for the positive microcontact printing (MCP) experiments. The high viscosity of the PMMA solution results in a slow dewetting process, leaving a large amount of PMMA residue in the OCT SAM covered regions. PEI was studied because the nitrogen atoms in the polymer chains could adsorb well to the gold surface. However, due possibly to the open dendrimer structure of the PEI network, the etchant ions can easily penetrate through the PEI layer[2]. As a result, the metal layer is almost completely removed. Table S1 summarizes the above experimental results and demonstrates that polymer backfilling molecules are not suitable for the roll-to-roll MCP process.

Table S1. Results of positive MCP using PMMA and PEI as the backfilling ink. (Scale bar, 100 μm).

Backfilling solution			After backfilling	After etching
Solute	Solvent	Concentration		
PMMA	n-Butyl acetate	80 mg/ml	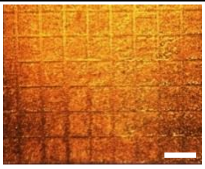	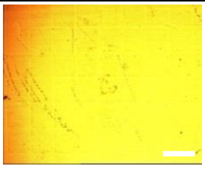
		40 mg/ml	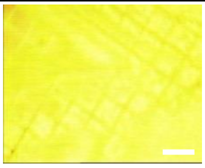	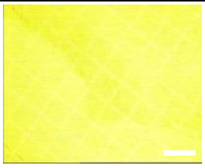
		10 mg/ml	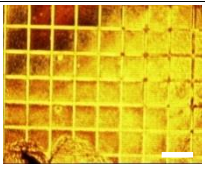	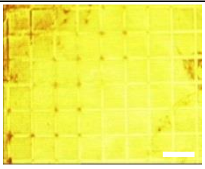
		5 mg/ml	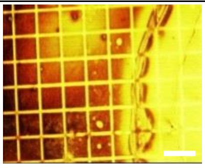	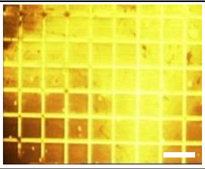
PEI	Diacetone alcohol/Ethanol (1:9, v/v)	10 mg/ml	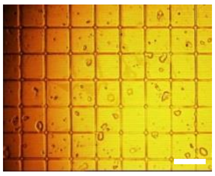	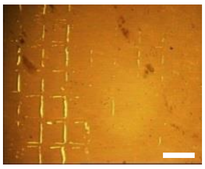

2. Microscale silver patterns

Microscale silver electrodes on flexible PET substrates were fabricated using the roll-to-roll MCP platform with a 184-PDMS mold. The rolling speed and printing pressure were 0.02 m s^{-1} and 15 N, respectively. As shown in Figure S1, silver mesh electrodes with sharp edges were obtained. A flexible P3HT:PCBM bulk heterojunction polymer solar cell (inset of Figure S1b) was fabricated using the silver mesh electrode as the anode. The current-voltage (I-V) curve of the solar cell under simulated AM 1.5 illumination is shown in Figure S1b, exhibiting a typical photovoltaic behavior.

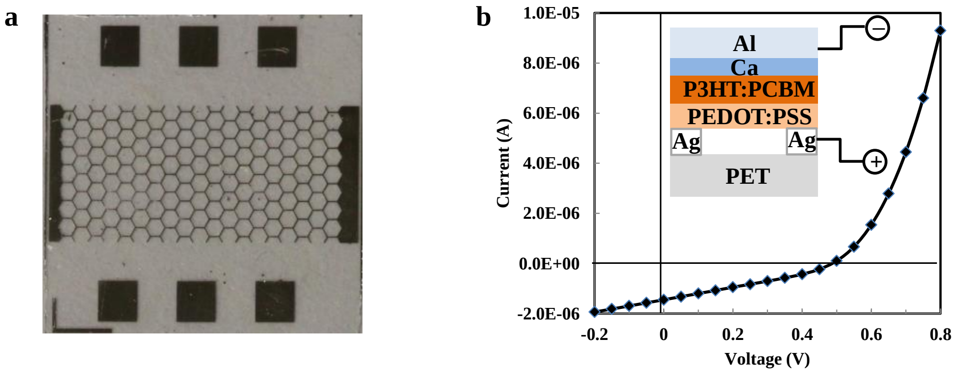

Figure S1. (a) Semitransparent silver hexgonal mesh electrodes (1.3 × 1.3 cm^2; linewidth: 40 μm, spacing: 600 μm) fabricated by the roll-to-roll MCP. (b) I-V curve of the PET/PEDOT:PSS/P3HT:PCBM/Ca/Al solar cell under illumination. The inset shows the device structure.

References

1. Benor, A., Gburek, B., Wagner, V. & Knipp, D. Organic transistors realized by an environmental friendly microcontact printing approach. *Organic Electronics* **11**, 831-835, (2010).
2. Perl, A., Péter, M., Ravoo, B. J., Reinhoudt, D. N. & Huskens, J. Heavyweight dendritic inks for positive microcontact printing. *Langmuir* **22**, 7568-7573, (2006).
